# Monitoring the Overall Quality of Groundwater Using a Geographic Information System in the Angads Plain (Oujda, Morocco)

**DOI:** 10.1155/tswj/7511804

**Published:** 2024-11-28

**Authors:** Latifa Taoufiq, Ilias Kacimi, Mohamed Saadi, Nordine Nouayti, Nadia Kassou, Karima El-Mouhdi

**Affiliations:** ^1^Geology Department, Geoscience, Water and Environment Laboratory, Faculty of Sciences, Mohammed V University in Rabat, Avenue Ibn Batouta, Rabat 10100, Morocco; ^2^Department of Civil, Environmental and Energy Engineering, Laboratory of Applied Sciences Water Management and Civil Engineering Team, National School of Applied Sciences Al Hoceima, Abdelmalek Essaadi University, Tetouan 32003, Morocco; ^3^Biology Department, Health and Environment Laboratory, Faculty of Science, Natural Resources Management and Development Team, Moulay Ismail University, Meknès, Morocco; ^4^Ministry of Health and Social Protection, Training Division, Higher Institute of Nursing and Health Techniques, Meknes, Morocco

**Keywords:** Angads aquifer, bacteriological, geographic information system, mapping, Morocco, overall quality, physicochemical

## Abstract

The future of groundwater is one of the key challenges for sustainable water management, hence the need to monitor its overall quality. The objective of this work is to assess the overall quality and determine the spatiotemporal evolution of the Angads aquifer in northeastern Morocco in 2014 and 2020, based on the parameters NH_4_^+^, NO_3_^−^, EC, Cl^−^, and FC, as well as the Geographic Information System (GIS). The results of the comparison of these five parameters between 2014 and 2020 show a general increase in NH_4_^+^ and a decrease in NO_3_^−^ and FC at most sampling points. These changes could be attributed to a shift in pollution sources or biological processes affecting water quality. On the other hand, the stability of EC and Cl^−^ levels suggests a consistency in the inputs of salts or minerals. The quality percentages show a decrease in good, poor, and very poor quality, following an increase in average quality, from 10.52% (in 2014) to 5.26% (in 2020), 31.57% (in 2014) to 21.05% (in 2020), 31.57% (in 2014) to 26.31% (in 2020), and 26.31% (in 2014) to 47.36% (in 2020), respectively. Spatial and temporal mapping of the quality over these 2 years shows that the deterioration continues toward the east, southeast, and southwest. This is justified by very high measurements of the parameters NO_3_^−^, EC, and Cl^−^ at sampling points 2, 3, 4, 5, 7, 8, and 15 for 2014 and 2020, reaching 156 mg/L, 10,570 µS/cm, and 3790 mg/L in 2014 and 134 mg/L, 10,355 µS/cm, and 3597 mg/L in 2020, respectively, due to effluents from pollution points such as the Oujda public landfill, the wastewater treatment plant, and the former Sidi Yahya landfill to the west. On the other hand, in the north, northeast, and northwest, there has been an improvement in quality due to the remoteness of these pollution points. In order to protect this vital resource, recommendations need to be put in place, in particular by treating leachates so as to ensure that the quality of the water is not discharged directly into the aquifer or used for other purposes, and to avoid discharging effluent from the wastewater treatment plant into the natural environment.

## 1. Introduction

Groundwater is an essential element for the life and sustainable development of a country. Morocco is at the limit of the stress threshold, which is around 950 m^3^/h/year of the resource. It will reach the threshold of shortage (500 m^3^/h/year) around 2030 [1]. The composition of groundwater reflects interactions with the characteristics of the soil it crosses, the plant cover, atmospheric inputs, the lithology of the geological formations that host the aquifers, and the presence of industrial or agricultural activities [2, 3].

The socioeconomic framework is divided into two components: social and economic. The social component has two aspects: on the one hand, education, which is particularly developed in the prefecture of Oujda-Angad, where our study area is located, with the largest number of schools in the entire region; on the other hand, healthcare, where the private sector is investing in infrastructure improvement and planning the opening of new clinics [4]. It is also important to highlight the significance of two major regional institutions El Farabi Hospital and the Psychiatric Hospital.

The second component, related to the economy, is divided into two parts: first, agriculture. The study area is characterized by low rainfall and has an exploitable agricultural area of approximately 103,517 ha, of which only 5.4% is irrigated. Next, industry, a growing sector in the Oujda-Angad prefecture, which includes more than 127 active units. These units are distributed across the following sectors: agri-food (33%), chemical and para-chemical (40.2%), metallurgical and mechanical (20.5%), textile and leather (3.6%), and finally electrical and electronic (2.7%) [4].

From a geographical perspective, the Angads aquifer is located in a subsiding basin, but a flexure divides it into two distinct units: the southern Angads, covering 250 km^2^, and the northern Angads, covering 200 km^2^. The transition between these two areas is characterized by a decrease in the thickness of the bedrock, accompanied by a rapid drop in elevation, which leads to a thickening of the aquifer near the airport. This results in a geometry forming two basins on either side of this flexure [5].

The deterioration in their quality due to high values of one or more physicochemical and bacteriological parameters caused by various sources of pollution, such as leachates from landfill sites, the intensive use of fertilizers and pesticides in agriculture, and the discharge of untreated wastewater, all constitute major dangers for these water resources and ecosystems [6].

The assessment of groundwater quality is the focus of several national and international studies using Geographic Information Systems (GISs) [7] and the Water Quality Index (WQI) [8] or other methods [9–11] as this is an important issue for the protection of human health and water consumption. In eastern Morocco, studies have been carried out on the quality of groundwater in the Angads aquifer in northeast Morocco [12], but they have not taken into account all the parameters and sources of pollution. This work holds particular significance as it complements existing studies by analyzing five key physicochemical parameters that determine the overall water quality in Morocco: electrical conductivity (EC), chloride ions (Cl−), nitrate ions (NO3−), ammonium ions (NH4+), and fecal coliforms (FC) for the years 2014 and 2020. The objective is to identify the parameters responsible for the degradation of groundwater quality. In addition, it highlighted the impact of two other major pollution factors, namely, the wastewater treatment plant (WWTP) and the Oujda public landfill, rather than a single factor, namely, the former Sidi Yahya landfill, on the quality of this groundwater [13].

The presence of these parameters with high values indicates the origin and type of water pollution; for example, EC is a good marker of water origin [14–18]. Moreover, to determine its origin, we need to know its mineralization. In fact, mineralization is the concentration of salts dissolved in water; the higher the concentration, the higher the mineralization. Additionally, EC is closely linked to mineralization, since it allows us to assess the quantity of dissolved salts and is considered a good indicator of the origin of the water [19]. A previous study carried out in the same study area [14, 20] showed, because of a multivariate statistical analysis of physicochemical parameters including EC, that the mineralization of this groundwater is controlled by the residence time in the aquifer. In addition, the nature of the geological formation it passes through, with limestone, more or less clayey silts and volcanic rocks from the Plio-Quaternary. NO_3_^−^ is released into the water by the leaching of nitrogen products from the soil by the decomposition of synthetic or natural fertilizers or organic matter [15]. Water-rich Cl^−^ is corrosive and laxative [19]. In reality, the concentration of Cl^−^ in water also depends on the terrain through which it flows [19]. NH_4_^+^ is the most abundant form of nitrogen in wastewater and is generated by industrial activities such as dairy farms and dairy piggeries [21]. On the other hand, it has an impact on health since the toxicity of NH_4_^+^ in water can lead to convulsions, death, and coma [22]. In addition, the presence of bacteria such as FC, total coliforms (TC), and fecal streptococci (FS) indicates fecal contamination [23]. The comparison of these five physicochemical and bacteriological parameters and the study of the overall quality of groundwater in the Angads aquifer in 2014 and 2020 are essential sources of information for characterizing the impact of agricultural, human, and industrial activities and the different landscape units [24]. It is in this context that the present work has been carried out to compare the values of these five parameters and to study the overall quality of the region's groundwater in 2014 and 2020 because of physicochemical and bacteriological analyses of the water, and to determine which areas are affected by degradation using the GIS.

## 2. Geological and Hydrogeological Setting of the Study Area

**Figure 1:**
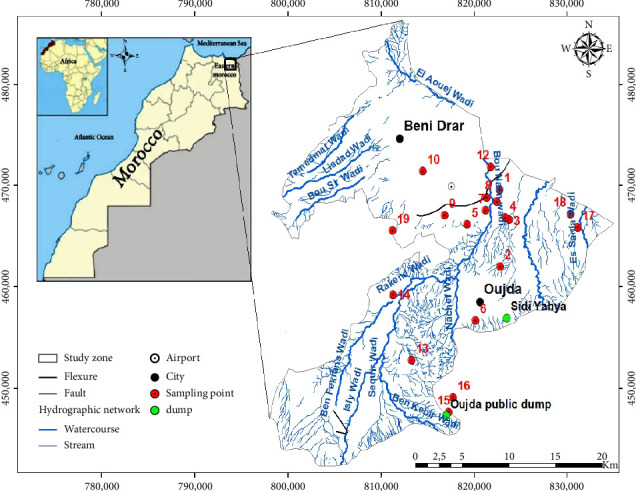
Angads aquifer and location of sampling points.

The Angads aquifer is located in northeast Morocco near the Algerian–Moroccan border (Figure 1). It is bounded to the south by the Jbel Hamra relief, to the east by the Marnia plain in Algeria, to the north by the Beni-Snassen range, and to the west by Jbels Megrez and Harraza that separate it from the Bou-Houria plain [14, 25].

**Figure 2:**
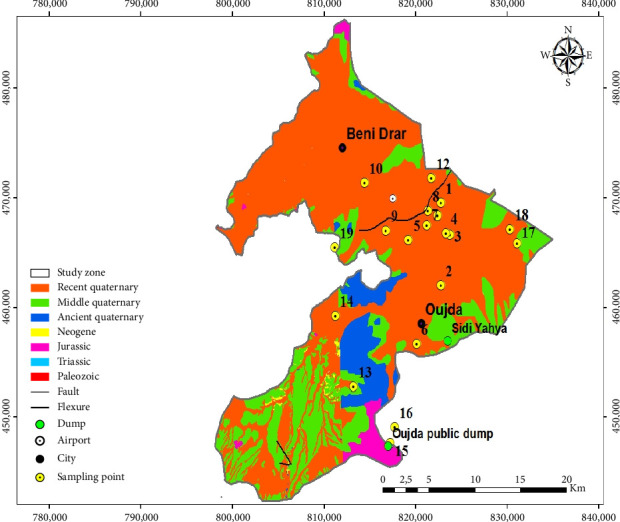
Geological map of the Angads aquifer [14].

From a geological point of view, the Angads aquifer (Figure 2) flows through formations composed essentially of more or less clayey silts, more or less calcareous encrusted soils, and volcanic rocks from the Plio-Quaternary, separated from the Lias dolomites by impermeable Miocene marl [26]. The Angads aquifer forms the eastern part of the Taourirt–Oujda corridor, this region being a subsiding basin since the Neogene [27].

In the hydrogeological context, this aquifer is characterized by a marly substratum. This aquifer consists of a subsidence basin in a graben system [28]. In the northern Angads (Figure 2), flow occurs from southwest to northeast through conglomerates, sands, alluvium, and clayey silts. In the southern Angads, the flow is from south to north in fissured or compact basalts, limestones, volcanic tuffs, conglomerates, alluvium, cinerites, silts, and sands [25].

## 3. Materials and Methods

### 3.1. Study Design and Sampling

This study builds on previous research, particularly the study on the impact of leachates from the former Sidi Yahya landfill in the city of Oujda, eastern Morocco, on groundwater quality [13]. The objectives of this work are to assess the impact of other pollution sources, namely, the discharges from the WWTP and the Oujda public landfill, on the groundwater of the Angads aquifer through comparative studies and quality assessments, as well as mapping the spatiotemporal evolution of water quality in 2014 and 2020. To achieve this, analyses were conducted on the five main physicochemical and bacteriological parameters of the simplified grid for evaluating overall groundwater quality according to Moroccan law. These parameters are as follows: (a) physical parameters, notably EC; (b) chemical parameters, such as nitrate ion (NO_3_^−^), ammonium ion (NH_4_^+^), and chloride ion (Cl^−^); and (c) bacteriological parameters, including FC. This study also uses the GIS tool to create the maps, following these steps: first, preparing the data, which includes collection and validation; second, data analysis; third, importing the data into GIS; fourth, creating the map by applying symbology and adding necessary elements (legend, scale, and title); fifth, validation and adjustment; and finally, sixth, exporting and sharing the map. These 2 years were chosen as they generally involve the same sampling points (wells and boreholes). Nineteen sampling sites were monitored in 2014 and 2020. These sites were chosen because they are the main sources of drinking water for the population and are located upstream and downstream of pollution points, namely, the WWTP and the Oujda public landfill. Groundwater sampling was carried out by the Moulouya Hydraulic Basin Agency in September 2014. To compare the results with those of 2020, the samples were collected during the same period and at the same sampling points, as follows: (a) six wells located at the WWTP: two upstream, represented on the map by Points 5 and 6, and four downstream, indicated by Points 7, 8, 11, and 12; (b) two wells near the Oujda landfill: one close to the landfill, designated by Point 15, and the other downstream, marked by Point 16; (c) two boreholes, identified by Points 13 and 19; and (d) the remaining points correspond to eleven wells scattered throughout the study area (Figure 1). The selected wells and boreholes were chosen because they primarily supply water to the population. The geographic coordinates of the structures were obtained using a GPS (GARMIN—GPSMAP64s).

Furthermore, two water samples were manually taken per well at all sampling points. The collected samples were placed in 1.5-L polyethylene bottles, which had been prewashed with distilled water and rinsed three times with groundwater. All samples were transported to the laboratory in coolers at 4°C. The physicochemical and bacteriological parameters were determined in situ and in the laboratory according to the protocol described by Rodier [29].

### 3.2. Analysis Method and Data Mapping

**Figure 3:**
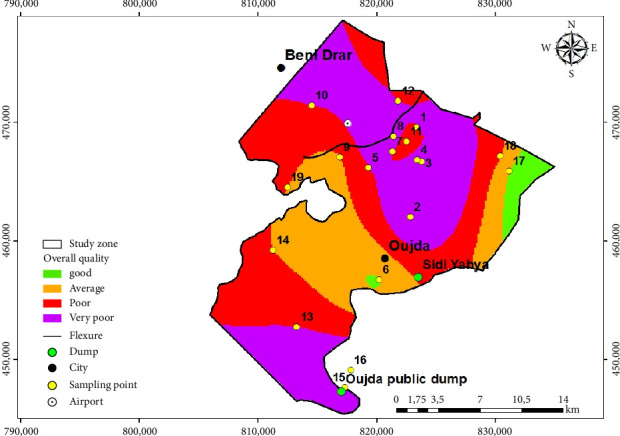
Map of the overall quality of the Angads aquifer in 2014.

**Figure 4:**
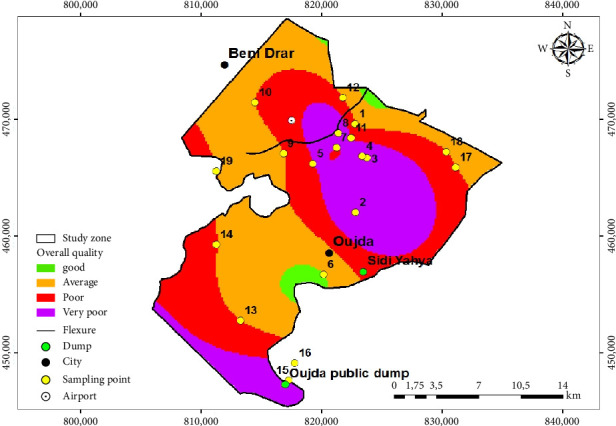
Map of the overall quality of the Angads aquifer in 2020.

The data collected for the five physicochemical and bacteriological parameters (EC, NO_3_^−^, NH_4_^+^, Cl^−^, and FC) from 19 sampling points were entered and grouped together in an Excel file. These data were processed and analyzed using descriptive statistics, calculating the maximum, minimum, mean, and standard deviation. The comparison of these five parameters for each sampling point was carried out manually in the form of diagrams using Excel, and an overall quality study based on the simplified groundwater quality table for Morocco, drawn up by the health authorities to protect public health and prevent illnesses linked to the consumption of polluted water [30] (Table 1). The design and production of maps for the spatiotemporal assessment of overall groundwater quality were carried out using the GIS, for the 2 years 2014 and 2020. They incorporate the interpolation of the coordinates of the structures (wells and boreholes), the former Sidi Yahya landfill, the Oujda public landfill, as well as the WWTP (Figures 3 and 4). In order to deduce the influence of the latter on the overall quality of this groundwater, the GIS enables data to be stored clearly and definitively, phenomena to be identified, risks to be anticipated, a wealth of attribute information on objects to be managed, maps to be created quickly, and locations to be pinpointed in time and space [31].

## 4. Results

### 4.1. Physicochemical and Bacteriological Analyses

The main results of the analysis of the five physicochemical and bacteriological parameters of the groundwater from 19 sampling points in the Angads aquifer in 2014 and 2020 are given in Table 2.

The results reveal that the highest value was recorded in EC, reaching up to 10,570 µS/cm in 2014 and 10,355 in 2020, while the lowest value was recorded in FC, which reached 0 UFC/100 mL in 2014 and 2020.

In fact, Cl^−^ values vary from 177 to 3790 mg/L with an average of 680 mg/L and a standard deviation of 844 in 2014, but in 2020, Cl^−^ values oscillate between 172 and 3597 mg/L, with an average of 607 mg/L and a standard deviation of 788 (Table 3).

In fact, the EC varies with time, since in 2014, it ranged from 1016 to 10,570 µS/cm with an average of 2597 µS/cm and a standard deviation of 2228, and for 2020, the EC varies from 986 to 10,355 µS/cm with an average of 2463 µS/cm and a standard deviation of 2171 (Table 3).

NO_3_^−^ values vary from 15 to 156 mg/L, with a mean of 59.8 mg/L and a standard deviation of 48 mg/L in 2014, and in 2020, NO_3_^−^ values vary from 5 to 134 mg/L with a mean of 50 mg/L and a standard deviation of 40 (Table 3).

NH_4_^+^ values vary from 0.009 to 0.108 mg/L, with a mean of 0.029 mg/L and a standard deviation of 0.03 in 2014. In 2020, NH_4_^+^ values ranged from 0.02 to 0.67 mg/L, with a mean of 0.07 mg/L and a standard deviation of 0.14 (Table 3).

FC values vary from 0 to 200,000 UFC/100 mL, with an average of 19,113 UFC/100 mL and a standard deviation of 4087.58 in 2014, while for 2020, FC values vary from 0 to 4800 UFC/100 mL with an average of 558 UFC/100 mL and a standard deviation of 1399 (Table 3).

Furthermore, according to Moroccan standards for human consumption, the physicochemical and bacteriological parameters, that is, EC, NH_4_^+^, Cl^−^, NO_3_^−^, and FC, must not exceed values of 2700, 0.5, 750, 50 mg/L, and 0 UFC/100 mL, respectively. Based on the results of this study, it can be deduced that the sampling points that exceed these standards are located downstream of the WWTP, and downstream and close to the Oujda public landfill for both 2014 and 2020.

### 4.2. Comparison of the Parameters EC, NO_3_^−^, Cl^−^, NH_4_^+^, and FC for the Nineteen Sampling Points in 2014 and 2020

**Figure 5:**
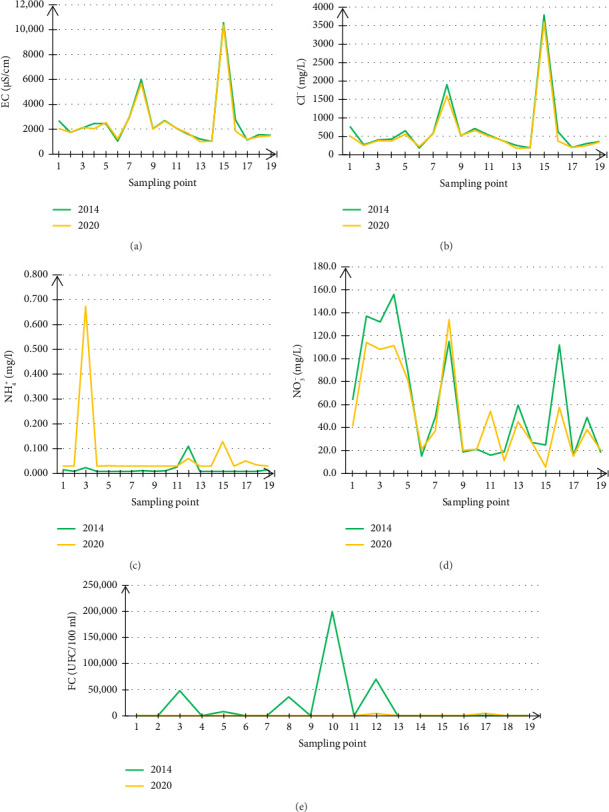
Comparison of variations in the values of the various physicochemical and biological parameters [EC (a), Cl^−^ (b), NH_4_^+^ (c), NO_3_^−^ (d), FC (e)] of the Angads groundwater in 2014 and 2020.

A comparative study generally requires the same sampling points over a given period, hence the choice of the two years 2014 and 2020. The results of the comparison of the five physicochemical and bacteriological parameters (EC, NO_3_^−^, Cl^−^, NH_4_^+^, and FC) of the groundwater from 19 points distributed over the Angads aquifer for these two years are illustrated in Figure 5.

The results for EC (Figure 5(a)) showed that the two curves were well correlated. These curves show an increase in EC values at sampling points 8 and 15 for both years, one of which is located downstream of the WWTP and the other near the Oujda public landfill. These points reached values of 5990 and 10,570 µS/cm, respectively, in 2014, while in 2020 they reached values of 5666 and 10,355 µS/cm, respectively. Additionally, these values far exceed the World Health Organization (WHO) value [32], which is around 400 mg/L. For the other sampling points, the values ranged from 1016 to 2950 µS/cm in 2014 and from 986 to 2903 µS/cm in 2020.

The monitoring of variations in the chloride ion (Cl^−^) in groundwater from 19 sampling points distributed over the Angads aquifer over the two years 2014 and 2020 is presented in Figure 5(b). The results show that the two Cl^−^ variation curves are well correlated. In fact, the increase in Cl^−^ was recorded at sampling points 8 and 15 for these 2 years, which are located one downstream of the WWTP and the other near the Oujda public landfill, respectively. These points reached values of 1897 and 3790 mg/L in 2014, respectively, whereas in 2020, they reached values of 1590 and 3597 mg/L, respectively. Furthermore, these values exceed the WHO value [32] of around 250 mg/L. On the other hand, for the other sampling points, Cl^−^ values varied between 177 and 763 mg/L in 2014, and in 2020, the values were between 172 and 656 mg/L.

The evolution of NH_4_^+^ ammonium ion in groundwater from 19 sampling points distributed over the Angads aquifer during the study years (2014 and 2020) is presented in Figure 5(c). The results show an increase in the NH_4_^+^ value in 2020 compared to 2014, particularly in the groundwater at sampling point 3, which is located in an agricultural environment, where the NH_4_^+^ value reached 0.67 mg/L in 2020 instead of 0.02 mg/L in 2014. For the other sampling points, NH_4_^+^ values varied between 0.007 and 0.10 mg/L in 2014 and between 0.02 and 0.13 mg/L in 2020. These values do not exceed the WHO guideline [32], which is around 1.5 mg/L.

In addition, Figure 5(d) illustrates the evolution of nitrate (NO_3_^−^) in groundwater at 19 sampling points distributed over the Angads aquifer over the two years 2014 and 2020. The results show that the two NO_3_^−^ variation curves are similar at the majority of the sampling points, with the highest values recorded at Points 1, 2, 3, 4, 5, 8, 13, and 16 for both years. These points are all located in agricultural areas and reached values of 64, 137, 132, 156, 89, 115, 59.4, and 112 mg/L, respectively, in 2014, and values of 42, 113, 107, 113, 82.5, 134, 46, and 58.5 mg/L, respectively, in 2020. However, for the other sampling points, NO_3_^−^ values varied between 15 and 49 mg/L in 2014 and between 5.38 and 55 mg/L in 2020. This means that these values exceed the value set by the WHO [32], which is around 50 mg/L.

Monitoring of bacteriological contamination, in particular, changes in FC in groundwater at 19 sampling points distributed over the Angads aquifer, over the course of 2014 and 2020, is illustrated in Figure 5(e). The results show an increase in the FC value of groundwater in 2014 compared with 2020, with this increase mainly affecting sampling points 3, 8, 10, and 12. The first two points are located in the agricultural environment and the last two points are located downstream of the WWTP, reaching values of 48,000, 36,000, 200,000, and 70,000 UFC/100 mL in 2014, respectively. In 2020, however, the FC values will reach 189, 79, 54, and 4201 UFC/100 mL, respectively.

### 4.3. Overall Quality of Angads Groundwater in 2014 and 2020

Determination of the overall quality of groundwater at 19 sampling points in the Angads aquifer in 2014 and 2020 is based on comparison of the values of five physicochemical and bacteriological parameters, namely, EC, Cl^−^, NO_3_^−^, NH_4_^+^, and FC with those of the simplified grid for quality assessment under Moroccan law [33].

When the values of these five parameters exceed the thresholds defined by this grid, it can affect groundwater quality and have potential health implications. Indeed, high EC reflects an increased concentration of dissolved ions in the water, such as salts (chlorides, sulfates, and bicarbonates), often due to saltwater intrusion or the excessive use of salts in agricultural and industrial activities [34]. Elevated EC can make the water potentially hazardous to health, particularly with prolonged consumption, contributing to kidney problems and electrolyte imbalances [35].

High nitrate concentrations in groundwater (above 50 mg/L) indicate contamination from anthropogenic sources [36]. Exposure to high levels of nitrates is linked to blue baby syndrome in infants, a condition where the blood cannot properly transport oxygen. Nitrates may also increase the risk of gastrointestinal cancers in adults [37].

Ammonium (NH_4_^+^) is often an indicator of recent contamination from organic matter or effluents [38]. Although ammonium is generally less toxic than nitrates, it can affect the sensory quality of water (taste and odor). Additionally, when converted into nitrates in the body, it presents similar health risks [35].

High chloride concentrations can indicate anthropogenic contamination [34]. Elevated chloride levels can make water salty and corrosive, thus affecting both health and the quality of drinking water. Furthermore, chloride-rich water can damage crops and agricultural soils [35].

FC signal biological contamination of water from fecal matter, often due to faulty sanitation systems or unsafe agricultural practices [39]. The presence of these bacteria indicates a high risk of waterborne diseases such as gastroenteritis and diarrhea. FC may also indicate the presence of other dangerous pathogens like *E. coli*, which can lead to serious diseases such as cholera and hepatitis [35].

The groundwater quality results for the Angads aquifer for the two years 2014 and 2020, presented in Table 4, show that in 2014, sampling points 2, 3, 4, 8, 15, and 16 were characterized by very poor quality, due to very high values of EC, Cl^−^, NO_3_^−^, and FC, which reached up to 10,570 µS/cm, 3790, 156 mg/L, and 48,000 UFC/100 mL, respectively. Sampling points 2, 3, and 4 are located in agricultural areas, point 8 is located downstream of the WWTP, Point 15 near the Oujda public landfill, and Point 16 downstream of this landfill. On the other hand, the groundwater at sampling points 1, 5, 7, 10, 12, and 13 is of poor quality, with EC, NO_3_^−^, and FC values of up to 2950 µS/cm, 89 mg/L, and 200,000 UFC/100 mL, respectively. In addition, sampling points 1, 10, and 13 are located in agricultural areas, while Point 5 is upstream of the WWTP and Points 7 and 12 are downstream of the plant. Sampling points 9, 11, 14, 18, and 19 are characterized by average quality, due to average values for the elements EC, Cl^−^, and NO_3_^−^, which reach values of 2056 µS/cm, 528, and 48.7 mg/L, respectively. Points 9, 14, 18, and 19 are located in agricultural areas, with the exception of Point 11, which is located downstream of the WWTP. With the exception of groundwater at sampling points 6 and 17, which are of good quality due to EC, NO_3_^−^, and FC values of 1110 µS/cm, 15.33 mg/L, and 310 UFC/100 mL, respectively, these points are located upstream of a WWTP and the other in an agricultural environment, respectively. Groundwater quality in the Angads aquifer in 2014 was 31.57% very poor quality, 31.57% poor quality, 26.31% average quality, and just 10.52% good quality.

In 2020, the quality of groundwater at sampling points 2, 3, 4, 8, and 15 was very poor, due to very high NO_3_^−^, EC, and Cl^−^ values of up to 134 mg/L, 10,355 µS/cm, and 3597 mg/L, respectively. The first three points are located in agricultural areas, Point 8 downstream of the WWTP and Point 15 near the Oujda public landfill. For groundwater, sampling points 5, 7, 11, and 16 are characterized by poor quality due to high NO_3_^−^ and EC values of up to 82.5 mg/L and 2903 µS/cm, respectively. Points 5 and 7 are located upstream of the WWTP, while Points 11 and 16 are located downstream of the WWTP. With the exception of the groundwater at sampling point 6, located upstream of the wastewater treatment plant, which exhibits good quality. However, the groundwater at sampling points 1, 9, 10, 12, 13, 14, 17, 18, and 19 was of average quality, with NO_3_^−^, EC, and Cl^−^ values of up to 46 mg/L, 2642 µS/cm, and 656 mg/L, respectively. These points are all located in agricultural areas, with the exception of Point 12, which is located downstream of the WWTP. The quality of groundwater in the Angads aquifer in 2020 is 26.31% very poor quality, 21.05% poor quality, 47.36% average quality, and barely 5.26% good quality.

**Figure 6:**
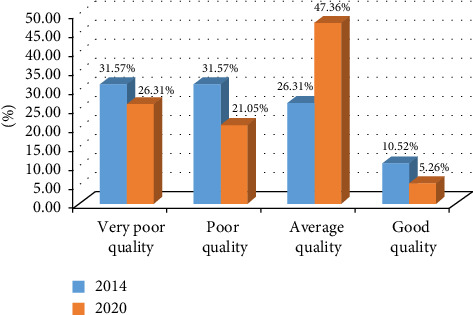
Percentage of overall groundwater quality in the Angads aquifer in 2014 and 2020.

In summary, the assessment of the overall quality of groundwater from 19 sampling points in the Angads aquifer is based on the five main parameters EC, Cl^−^, NO_3_^−^, NH_4_^+^, and FC of the simplified grid for quality assessment under Moroccan law in 2014 and 2020 with rates of variation that vary by −5%, −11%, −15%, 371%, and −97%, respectively. This rate is generally the relative variation in a value between two given periods. It is calculated as a percentage of the difference between the average annual value of some of these parameters in 2014 and 2020. To make it a relative value, we divide it by the average annual value for 2014, to obtain a value in decimal numbers, and if we want to reduce it to a percentage, we can display it as a percentage in Excel. The percentages of good, poor, and very poor-quality water decreased during this period, from 10.52% (in 2014) to 5.26% (in 2020), 31.57% (in 2014) to 21.05% (in 2020), and 31.57% (in 2014) to 26.31% (in 2020). On the other hand, the percentages of medium-quality water have been increased from 26.31% (in 2014) to 47.36% (in 2020) (Figure 6).

### 4.4. Spatiotemporal Evolution of the Distribution of the Five Physicochemical and Bacteriological Parameters and the Overall Quality of Groundwater in the Angads Aquifer in 2014 and 2020

The spatial and temporal evolution of the overall quality of groundwater at 19 sampling points distributed over the Angads aquifer in 2014 and 2020, focusing on areas where data are available, is illustrated in Figures 3 and 4. These maps were drawn up using a GIS tool, by interpolating the quality families for the 19 sampling points, and the coordinates of (a) the structures (wells and boreholes), (b) the Oujda public landfill site, (c) the WWTP, and (d) the former Sidi Yahya landfill site. In order to make a comparative study and quality for these 2 years, the results reveal, on the one hand, an improvement in the quality of these waters in the north, northeast, and northwest, ranging from very poor and poor quality to average quality in 2014 and 2020. On the other hand, in the east, south, southeast, and southwest, where the Oujda public landfill site is located, the former Sidi Yahya landfill site (in the commune of Sidi Yahya), and upstream and downstream of the WWTP, the quality of this groundwater has not improved, as it remains between poor and very poor, and so, the deterioration has continued in 2014 and 2020.

**Figure 7:**
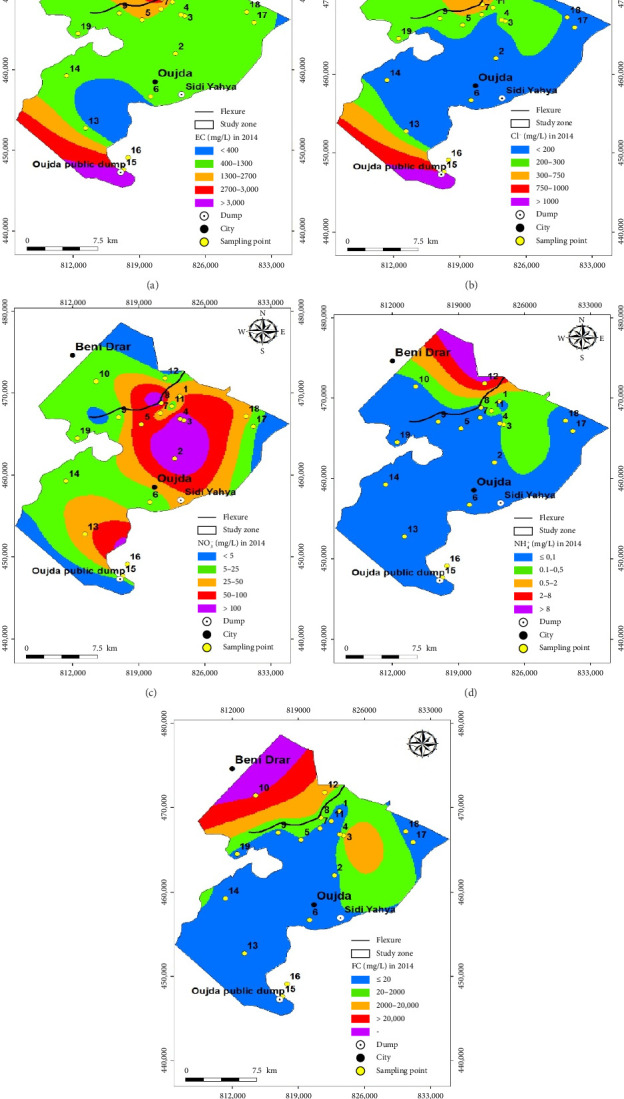
Map of the spatial and temporal distribution of physicochemical and bacteriological parameters in 2014.

**Figure 8:**
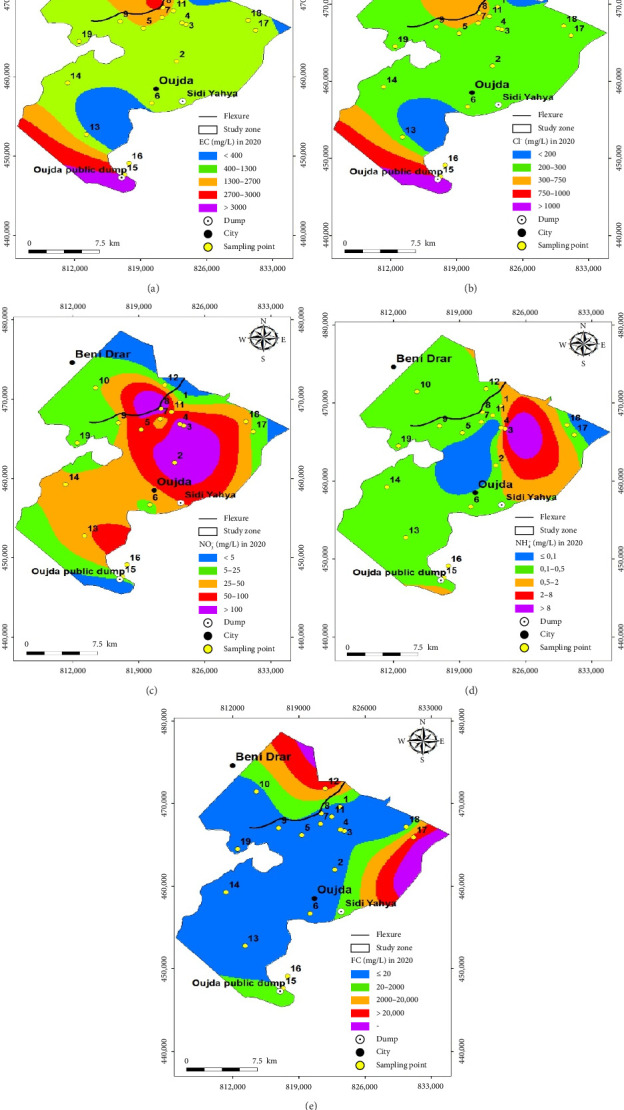
Map showing the spatial and temporal distribution of physicochemical and bacteriological parameters in 2020.

Figures 7 and 8 illustrate the maps of spatiotemporal variation in the values of the five physicochemical and bacteriological parameters, namely, EC, Cl^−^, NH_4_^+^, NO_3_^−^, and FC, in the groundwater from 19 sampling points in the Angads aquifer over two years in 2014 and 2020. In fact, during the two years of study, the results showed that the EC values in 19 sampling points showed a slight decrease in values in the northeast of the study area, reflecting an improvement in the quality of these waters (Figures 7(a) and 8(a)). While Cl^−^ values increased in the central, eastern, southeastern, and southwestern parts of the study area, we indicate that it has had an impact on quality, moving from excellent to good (Figures 7(b) and 8(b)). For the other zones, the values remain broadly the same.

In addition, the NH_4_^+^ results show a more significant increase in the center and southeast of the study area, revealing that there has been an influence on water quality, which has gone from excellent to good and sometimes average (Figures 7(d) and Figure 8(d)). However, NO_3_^−^ values have increased, particularly in the center of the study area, which shows that there has been an impact on the quality of these waters, which has gone from good to average (Figures 7(c) and Figure 8(c)). FC values in groundwater have increased, particularly in the east and southeast, indicating that the quality has changed from excellent to good, average, or even poor (Figures 7(e) and 8(e)).

## 5. Discussion

Given the importance of the overall quality of groundwater for human consumption worldwide, water quality studies are an essential element in the rational management of water resources and a tool for decision-makers in the implementation of sustainable development plans [40–42]. The aim of this study is to compare the values of five physicochemical and bacteriological parameters, such as EC, Cl^−^, NH_4_^+^, NO_3_^−^, and FC, and to study the overall quality of 19 sampling points in the Angads aquifer in northeast Morocco on the basis of the parameters of the simplified groundwater quality grid, in accordance with Moroccan law, in 2014 and 2020.

To determine whether the quality has improved or deteriorated over these two years. On the other hand, analyze the spatiotemporal evolution of the overall quality and physicochemical parameters of this aquifer using a GIS during this period, in order to identify the areas most affected by degradation and determine the causes responsible for this deterioration.

Analysis of the results of the comparison of these physicochemical and bacteriological groundwater parameters from 19 sampling points spread across the Angads aquifer in 2014 and 2020 reveals that EC and Cl^−^ values are virtually the same in the majority of sampling points in 2014 and 2020. There is therefore a relationship between EC and Cl^−^ [43], which varies according to the geological terrain traversed [44]. EC reflects the mineralization and origin of the water [45]. In fact, measuring conductivity makes it possible to assess the quality of the salts dissolved in the water [44]. Cl^−^ is an inorganic ion contained in the salt dissolved in the water, namely, potassium salt (KCl) and sodium salt (NaCl) [46]. Lithology also provides information on the composition of sediments and rocks [47], and generally influences EC, since the higher the EC, the more ions the solution will contain, from the minerals that make up the rock [48].

In our study area, the highest EC and Cl^−^ values were recorded at sampling points located downstream of the WWTP and near the Oujda public landfill. These values far exceed the WHO forecasts [32]. These results confirm the influence of infiltration of landfill leachate and effluent from the treatment plant, as well as a lithological influence. In fact, the impact of landfill leachate on groundwater quality has been confirmed by previous studies, such as the one carried out in the city of Sulaymaniyah in Iraq using the Tanjaro open-cast landfill, which revealed the effect of certain parameters exceeding WHO standards (EC, zinc (Zn) and iron (Fe), total dissolved solids, and total hardness) found in groundwater in the region [49]. The same is true of the study carried out on the groundwater in the Sfax-Agareb aquifer (southeast Tunisia) [50], which shows the harmful impact of leachates on the quality of this water, mainly in the downstream part of this aquifer, particularly in terms of zinc (Zn) and aluminum (Al).

Chloride (Cl^−^) has also been studied by several researchers, including Manasreh and his colleagues [51], because of an analysis of the chemical parameters of treated wastewater from the Al-Lajoun WWTP in Jordan. The result showed that this wastewater contained a high concentration of this element, exceeding the standards set in the Jordanian guidelines recommended for discharging water into valleys. This result is similar to our study analyzing (Cl^−^). The effects of effluent can explain this from WWTPs.

On another front, NH_4_^+^ monitoring showed an increase from 2014 to 2020. This may be due to industrial waste such as manganese electrolytic residues [52, 53], other social activities, contaminated land [54], livestock effluents [55, 56], and agricultural nitrogen fertilizers [57]. However, the NH_4_^+^ values found in the study area did not exceed the WHO standards [32] of 1.5 mg/L and therefore do not constitute a risk for the population. The same study by Abouelouafa and colleagues [58] showed that wastewater from the town of Oujda contained the lowest value of ammoniacal nitrogen. This could be explained by the oxidation of organic matter leading to an increase in ammoniacal nitrogen.

For changes in (NO_3_^−^) in groundwater in the study area over the two years 2014 and 2020, the results show that the trends of the two curves are similar at the majority of sampling points, with the highest values recorded at sampling points in agricultural areas, which exceed the value determined by WHO standards [32]. These results are consistent with a national study specifically on groundwater in the northern part of the town of Settat (Morocco) using the same analytical approach, carried out by El Mostafa Hassoune and colleagues [59], which revealed chemical pollution by the element nitrate due to runoff from the Oued Boumoussa. This is justified by nitric pollution. Similarly, scientific evidence has shown that the increase in NO_3_^−^ is due to the intensive use of fertilizers in agriculture, confirmed by a previous study carried out on groundwater in Kansas [60].

In terms of changes in the FC value of Angads groundwater in 2014 compared with 2020, the results show an increase in 2014 which mainly affects sampling points 3, 10, 8, and 12, the first two of which are located in agricultural areas, while the last two are located downstream of the WWTP. These points reached values of 48,000, 200,000, 36,000, and 70,000 UFC/100 mL, respectively, in 2014, while in 2020, these points will reach 189, 54, 79, and 4201 UFC/100 mL. An increase in the FC value most often reflects contamination of fecal origin. Previous studies have shown, including the first study on the quantification of animal and human viruses to differentiate the origin of fecal contamination [61], which revealed that microbiological indicators such as enterococci, *Escherichia coli*, and FC are the most commonly analyzed to assess the level of fecal contamination. The second in the town of Oujda (Morocco) by Aboulouafa et al. [58] revealed that the concentrations of FS and FC were very high and exceeded the WHO recommendations for water intended for nonrestrictive irrigation, which are of the order of 1/100 mL. The presence of these germs is an indicator of fecal contamination.

According to these earlier studies, the decrease in NO_3_^−^ and FC values in 2014 compared with 2020 can therefore be explained by a reduction in the use of fertilizers and feces, respectively, due to the decline in agriculture and livestock farming, and consequently the protection of the study area from agricultural pollution.

The analysis of the study results evaluating the overall groundwater quality of the Angads aquifer for 2014 and 2020 showed a decrease in the percentage of good-quality water at certain sampling points, indicating a degradation in water quality. These findings are consistent with previous studies conducted in the same study area, notably the one carried out by El and colleagues in 2013 [13]. Their results revealed a significant decline in water quality, with high concentrations of iron, zinc, and nickel, far exceeding French groundwater standards, attributed to the impact of leachates from the former Sidi Yahya public landfill. Furthermore, a study conducted by Rachid in 2015 [25], monitoring nitrate levels in this water through 17 piezometers, revealed an increase in nitrate concentrations between 1991 and 2008, confirming a progressive deterioration in water quality in the studied area.

These results are similar to previous studies; in particular, the study carried out on the groundwater of the Jurassic aquifers of the upper Ziz basin (Central High Atlas, Morocco) by Nouayti, Khattach, and Hilali [62], which shows that the overall quality of the groundwater in this aquifer varies according to the sampling points, ranging from good, average, and poor quality. In other regions of Morocco, such as Meknes and mainly the waters of the Ain Salama-Jerri spring, the EC and Cl^−^ values are 2000 µS/Cm and 532.5 mg/L, respectively, which is very high compared with Moroccan standards (NM) 03.07.001/2006 [63]. Similarly, the study carried out on the surface waters of the Moulouya wadi by Makhoukh and colleagues [46] reveals a deterioration in the quality of these waters, mainly in the area where wastewater from the town of Missouri is discharged, with high Cl^−^ and EC values of up to 750 mg/L and 3920 µs/cm, respectively, exceeding the Moroccan standard for surface waters [64].

On the other hand, the results of the maps of the spatiotemporal evolution of the overall quality of the water in the Angads aquifer in northeast Morocco in 2014 and 2020, using a GIS, show on the one hand an improvement in the quality of this water in the north, northeast, and northwest parts. On the other hand, the deterioration continues in the east, south, southeast, and southwest, where the Oujda public landfill and the former Sidi Yahya landfill (in the commune of Sidi Yahya) are located. This deterioration is due to the influence of leachates from these landfills, which are a source of pollution and result from the percolation of runoff and rainwater through the waste [65]. According to Pronost and Matejka [66], the compositions of the leachates are very different, with mineral compounds such as Ca^2+^, Mg^2+^, Na^+^, K^+^, NH_4_^+^, Fe^2+^, HCO_3_^−^, Cl^−^, and SO_4_^2−^. This confirms the influence of leachates on groundwater in the Angads aquifer, with high values for the parameters EC, Cl^−^, NH_4_^+^, and NO_3_^−^, which exceed national standards for human consumption [67] and those of the WHO [32]. These results are similar to previous studies; in particular, the study of groundwater contamination by landfill leachate (Etueffont, Belfort, France), by Khattabi et al. [68], reveals high concentrations of most parameters, mainly in wells located near the landfill. These parameters are hydrogen potential (pH), temperature (T°), EC, chloride ion (Cl^−^), oxidation–reduction potential (Eh), oxygen (O_2_), sulfate ion (SO_4_^2−^), nitrate ion (NO_3_^−^), calcium ion (Ca^2+^), sodium ion (Na^+^), copper ion (Cu^2+^), magnesium ion (Mg^2+^), potassium ion (K^+^), manganese ion (Mn^2+^), nickel ion (Ni^2+^), zinc ion (Zn^2+^), and total iron. These factors threaten the overall quality of the groundwater in the Angads aquifer and consequently the health of the population and the environment.

To preserve this essential resource, it is necessary to adopt effective pollution control strategies and sustainable groundwater management practices. This includes Integrated Water Resources Management [69], which is based on the coordination of surface and groundwater, stakeholder involvement, and the implementation of appropriate legislative frameworks. Reducing agricultural pollution requires the adoption of more sustainable practices, such as limiting the use of fertilizers and pesticides, and establishing vegetated buffer zones [70]. Protecting catchment areas is also crucial, with the creation of protected zones around drinking water wells to prevent contamination and restrict high-risk activities [71]. The treatment and reuse of wastewater are also critical, allowing it to be used for irrigation or aquifer recharge, while adhering to strict quality standards [72]. Additionally, participatory governance encourages the engagement of local communities in the management and protection of water resources [73]. Finally, monitoring with GISs allows for real-time tracking of water withdrawals and quality [74].

## 6. Conclusion

This study is an assessment of the overall groundwater quality of the Angads aquifer (Oujda, Morocco), based on the analysis of physicochemical and bacteriological parameters (NH_4_^+^, NO_3_^−^, EC, Cl^−^, and FC) and its spatiotemporal evolution using GIS for the 2 years 2014 and 2020. The main results obtained during this period revealed an increase in NH_4_^+^ and a decrease in FC and NO_3_^−^ at most sampling points. These changes could be attributed to a shift in pollution sources or biological processes. These factors are affecting groundwater quality, with ongoing spatiotemporal degradation in the eastern, southern, southeastern, and southwestern areas of the study zone, linked to discharges from pollution sources such as the WWTP, the Oujda public landfill, and the former Sidi Yahya landfill to the west. This study suggests a current vulnerability assessment to define protection perimeters, integrated water resource management, the reduction of agricultural pollution, and the integration of artificial intelligence for future forecasting.

## Figures and Tables

**Table 1:** Parameters and classes of the simplified grid for assessing the overall quality of groundwater [27].

Quality parameter	EC (*μ*s/cm)	Cl^−^ (mg/L)	NO_3_^−^ (mg/L)	NH_4_^+^ (mg/L)	FC (UFC/100 mL)
Excellent	< 400	< 200	< 5	≤ 0.1	≤ 20
Good	400–1300	200–300	5–25	0.1–0.5	20–2000
Average	1300–2700	300–750	25–50	0.5–2	2000–20000
Poor	2700–3000	750–1000	50–100	2–8	> 20,000
Very poor	> 3000	> 1000	> 100	> 8	—

*Note:* Source: Moulouya Hydraulic Basin Agency of Oujda Angads, Morocco.

**Table 2:** Results of physicochemical and bacteriological analyses of the Angads aquifer in 2014 and 2020.

Sampling points	EC (*μ*S/cm)		Cl^−^ (mg/L)		NO_3_^−^ (mg/L)		NH_4_^+^ (mg/L)		FC (CFU/100 mL)	
	2014	2020	2014	2020	2014	2020	2014	2020	2014	2020
1	2670	2040	763	503	64	42	0.014	0.029	0	41
2	1725	1721	265	248	137	113	< 0.007	0.028	40	34
3	2085	2139	390	372	132	107	0.022	0.673	48,000	189
4	2440	2019	419	369	156	113	< 0.007	0.029	95	111
5	2435	2521	645	549	89	82.5	< 0.007	0.03	8000	44
6	1030	1201	177	215	15	20	< 0.007	0.029	0	31
7	2950	2903	578	561	49	38	< 0.007	0.029	0	3
8	5990	5666	1897	1590	115	134	0.01	0.029	36,000	79
9	2030	1992	507	516	18.6	19	< 0.007	0.029	0	121
10	2680	2642	706	656	21	20	0.009	0.029	200,000	54
11	2056	2076	528	496	15.8	55	0.024	0.029	4	64
12	1580	1651	379	386	18.7	10	0.108	0.05	70,000	4201
13	1195	986	248	172	59.4	46	< 0.007	0.029	0	0
14	1016	1031	181	175	26.7	26	< 0.007	0.029	0	7
15	10,570	10,355	3790	3597	24.8	5.38	< 0.007	0.13	0	601
16	2750	1850	620	366	112	58.5	< 0.007	0.029	200	188
17	1110	1152	191	192	15.3	16	< 0.007	0.05	310	4800
18	1547	1390	291	238	48.7	38	< 0.007	0.033	500	1
19	1500	1466	351	337	18.1	19	0.014	0.03	0	31

**Table 3:** Values for physicochemical and bacteriological parameters in the Angads aquifer in 2014 and 2020.

Parameters	EC (*μ*S/cm)		Cl^−^ (mg/L)		NO_3_^−^ (mg/L)		NH_4_^+^ (mg/L)		FC (UFC/100 mL)	
	2014	2020	2014	2020	2014	2020	2014	2020	2014	2020
Maximum	10,570	10,355	3790	3597	156	134	0.108	0.67	200,000	4800
Minimal	1016	986	177	172	15	05	0.009	0.02	0	0
Average	2597	2463	680	607	59.8	50	0.029	0.07	19,113	558
Standard deviation	2228	2171	844	788	48	40	0.03	0.14	48,087.58	1399

**Table 4:** Overall quality of the Angads aquifer for 2014 and 2020 [33].

Sampling points	EC (*μ*S/cm)		Cl^−^ (mg/L)		NO_3_^−^ (mg/L)		NH_4_^+^ (mg/L)		FC (UFC/100 mL)		Overall quality	
	2014	2020	2014	2020	2014	2020	2014	2020	2014	2020	2014	2020
1	2670	2040	763	503	64	42	0.014	0.029	0	41	Poor	Average
2	1725	1721	265	248	137	113	< 0.007	0.028	40	34	Very poor	Very poor
3	2085	2139	390	372	132	107	0.022	0.673	48,000	189	Very poor	Very poor
4	2440	2019	419	369	156	113	< 0.007	0.029	95	111	Very poor	Very poor
5	2435	2521	645	549	89	82.5	< 0.007	0.03	8000	44	Poor	Poor
6	1030	1201	177	215	15	20	< 0.007	0.029	0	31	Good	Good
7	2950	2903	578	561	49	38	< 0.007	0.029	0	3	Poor	Poor
8	5990	5666	1897	1590	115	134	0.01	0.029	36,000	79	Very poor	Very poor
9	2030	1992	507	516	18.6	19	< 0.007	0.029	0	121	Average	Average
10	2680	2642	706	656	21	20	0.009	0.029	200,000	54	Poor	Average
11	2056	2076	528	496	15.8	55	0.024	0.029	4	64	Average	Poor
12	1580	1651	379	386	18.7	10	0.108	0.05	70,000	4201	Poor	Average
13	1195	986	248	172	59.4	46	< 0.007	0.029	0	0	Poor	Average
14	1016	1031	181	175	26.7	26	< 0.007	0.029	0	7	Average	Average
15	10,570	10,355	3790	3597	24.8	5.38	< 0.007	0.13	0	601	Very poor	Very poor
16	2750	1850	620	366	112	58.5	< 0.007	0.029	200	188	Very poor	Poor
17	1110	1152	191	192	15.3	16	< 0.007	0.05	310	4800	Good	Average
18	1547	1390	291	238	48.7	38	< 0.007	0.033	500	1	Average	Average
19	1500	1466	351	337	18.1	19	0.014	0.03	0	31	Average	Average

## Data Availability

The data used in this study are included within the content of the article.
